# The role of sexual mindfulness in enhancing sexual well-being among breast cancer survivors: A randomized clinical trial

**DOI:** 10.1097/MD.0000000000051237

**Published:** 2026-10-23

**Authors:** Asefeh Shirdashtzadeh Kerdabadi, Zeinab Tavakol, Fereshteh Rasti Borujeni, Hadi Raeisi Shahraki, Zahra Davoodi

**Affiliations:** a Department of Midwifery, Nursing and Midwifery School, Shahrekord University of Medical Sciences, Shahrekord, Iran; b Department of Reproductive Health, Community‑Oriented Nursing Midwifery Research Center, Nursing and Midwifery School, Shahrekord University of Medical Sciences, Shahrekord, Iran; c Department of Epidemiology and Biostatistics, School of Public Health, Shahrekord University of Medical Sciences, Shahrekord, Iran; d Family Counseling, Faculty of Medicine, Shahrekord University of Medical Sciences, Shahrekord, Iran.

**Keywords:** breast cancer, sexual mindfulness, sexual well-being

## Abstract

**Background:**

Patients with a wide range of sexual disorders are vulnerable to negative sexual well-being due to complications from breast cancer treatment. This study aimed to investigate the impact of sexual mindfulness-based training on the sexual well-being of women with breast cancer.

**Methods:**

This randomized clinical trial was conducted in 2024 and involved a statistical population of women with breast cancer who had consulted comprehensive health service centers in Shahrekord, Iran. Among the referring women, 36 people were selected by convenience sampling. To investigate depression in the participants, the short-form Beck Depression Questionnaire was used, and 4 patients were excluded from the study due to severe depression. Then, the participants were randomly assigned to 2 study groups of 16 patients.

Sexual mindfulness training was conducted in 8 sessions (90 minutes, once a week) for the intervention group at the reproductive and sexual health clinic in Shahrekord. Meanwhile, the control group was not subjected to planned intervention or training. A sexual well-being questionnaire was used to collect data for repeated measures analysis. Both groups were asked to complete the sexual well-being questionnaire before, immediately after, and 3 months after the intervention. Data were analyzed through independent samples *T*-test, analysis of covariance, and repeated measures analysis.

**Results:**

The findings showed that there was no significant difference in sexual well-being between the intervention and control groups before the intervention (*P* = .29). Immediately after the intervention, sexual well-being significantly increased in the intervention group, from 39.33 ± 25.56 to 56.17 ± 22.29 (*P* < .001); however, the corresponding between-group comparison was not statistically significant (*P* = .11). Three months after the intervention, sexual well-being was significantly higher in the intervention group (66.42 ± 13.79) than in the control group (51.92 ± 20.62; *P* < .001).

**Conclusion:**

Sexual Mindfulness can be considered a therapeutic method in palliative care to improve sexual well-being in breast cancer patients.

## 1. Introduction

The threat of breast cancer to human life is a worrying event, as it is the most common type of cancer and the primary cause of cancer-related death among women worldwide.^[1]^

Previous projections based on the Iranian National Population-Based Cancer Registry estimated that the number of breast cancer cases in Iran would reach 25,013 by 2025, representing a projected 63.0% increase in incidence among Iranian women.^[2]^

Today, the number of breast cancer survivors is rising due to the advancement of community-based screening programs and therapies. The 5-year survival rate for individuals with cancer has reached around 76% to 92%.^[3]^ According to research, breast cancer has a significant impact on sexual disorders. Conventional treatments such as surgery, radiotherapy, chemotherapy, hormone therapy, and anticancer drugs are effective for dealing with a variety of sexual issues.^[4-6]^ Various pieces of evidence indicate that the diagnosis and treatment of breast cancer can disrupt each of the components of sexual health, leading to a wide range of sexual problems in patients. This results in sexual disorders reported at a prevalence of approximately 73% among all age groups of breast cancer survivors.^[7]^ If sexual concerns persist for a long time, they can affect the satisfaction of sex and sexuality in patients with breast cancer.^[8,9]^

It has been proven in studies that sexual well-being is a key factor in determining satisfaction and quality in marriage.^[10]^ This approach to sexual health is comprehensive and encompasses both physical and mental aspects. Various components encompass sexual interest, intimate and healthy relationships, functioning, and sexual satisfaction.^[11-13]^

The results of the Oberguggenberger et al study showed that a change in sexual well-being is considered one of the most destructive aspects of life after successful breast cancer treatment.^[14]^ The negative consequences of cancer treatment, with great challenges such as negative attitudes and turbulent imagery, include a fear of touching in relationships and sexual attractions in women, making it difficult for these people to enjoy sexual interactions.^[15,16]^

However, in the healthcare system, with greater emphasis on continuing the patient’s life, less attention is paid to the sexual health of the patients and typically, it fails to provide satisfactory results in addressing sexual gaps in this group of patients.^[17]^ Moreover, feelings of shame and resistance among both patients and healthcare providers when discussing concerns related to sexual health and sexual issues are seen as barriers to patients accessing palliative care and counseling in the context of sexuality.^[10]^

Mindfulness, originally introduced by Kabat-Zinn, has emerged as an important therapeutic approach in supportive cancer care.^[18]^ It is a kind of meditation of presence in the present moment. By focusing on feelings and self-exploration, it causes the development of skills in people, leading to a reduction of stress and rumination.^[19-21]^

Sexual mindfulness is a subtype of mindfulness specifically considered in relational and sexual consequences and consists of 2 dimensions, Sexual Mindfulness and nonjudgment during a sexual experience, which makes people through the presence be completely freed from marital sexual conflicts in their sexual interactions.^[22]^ Sexual mindfulness teaches participants specific guidelines and, using mindfulness techniques, focuses attention on the body and sensed communications during sexual interactions. Examples include paying attention to breathing during intercourse, embracing, being aware of the sensations of touching and being touched by a partner, and being conscious of one’s own body and that of the partner, especially each other’s sexual organs,^[22,23]^ which leads to a release from intrusive thoughts and unwanted feelings during sexual encounters.^[24,25]^ Research in this area has shown that individuals who learn sexual mindfulness techniques and the components of a healthy sexual relationship gain a better understanding of each other’s sexual needs, which in turn fosters greater empathy and compassion in relationships, leading to increased satisfaction in marital relations.^[23,26]^

Leavitt has shown in his research that sexual mindfulness, by using mindfulness practices and sexual skills training, leads to the improvement of certain individual indicators in sexual relationships and is related to higher sexual satisfaction and self-esteem in sexual activities.^[27]^

Another study suggests that being sexually alert can help women focus on their sexual pleasure, which in turn increases their sexual satisfaction.^[28]^ This issue is significant for women because, on the 1 hand, they often place more emphasis on their partner’s arousal, and on the other hand, women who are more aware tend to be less anxious during sexual relations and are more likely to experience orgasm.^[29]^ The results of most studies examining mindfulness characteristics have shown that individuals with lower levels of mindfulness report lower sexual desire and more sexual dissatisfaction in their sexual interactions, which in the long run has been linked to feelings of dissatisfaction with sexual relationships and negative psychological and social consequences in married life.^[24,30]^

Given that sexual disorders are common adverse consequences of cancer treatment,^[4,5]^ and that no previous study has investigated the application of sexual mindfulness to improve sexual health among breast cancer survivors, the present study was conducted to investigate the impact of sexual mindfulness techniques on the sexual well-being of breast cancer survivors.

## 2. Materials and methods

This research employed a randomized clinical trial using a pretest–posttest design with a control group, which was conducted in 2024. The statistical population consisted of all women with breast cancer referring to selected comprehensive health service centers located in Shahrekord. These centers were chosen by a 2-stage cluster sampling method. After preparing a list of comprehensive health centers in Shahrekord city, each center was considered as a cluster, and 3 centers were randomly selected from among 8 clusters.

The sample size was determined based on the study by Soleimani. Taking into account the mean difference of 5.7 and the standard deviation of 3 for the variable of Sexual Function, as well as the type 1 error (α = 0.01) and the type 2 error (β = 0.05), the required sample size was calculated as 10 patients for each group. To account for possible dropouts, 18 patients were considered for each intervention and control group.^[31]^


$$N=\frac{\left(\overset{2}{\underset{1}{S}}+\overset{2}{\underset{2}{S}}\right){\left(Z_{1-\frac{\alpha }{2}}+Z_{1-\beta }\right)}^{2}}{{\left(\mu_{1}-\mu_{2}\right)}^{2}}$$

$$\begin{array}{c} \frac{\left(9+9\right){\left(2.58+1.64\right)}^{2}}{5.7^{2}}=\frac{320.55}{32.49}=9.9 \end{array}$$

After obtaining approval from the Ethics Committee of Shahrekord University of Medical Sciences (IR.SKUMS.REC.1402.060), the researcher informed the relevant officials at each center about the study’s objectives. With their permission, sample collection was conducted from February to March 2024. Then, 36 women were selected from the attendees using a convenience sampling method. All participants signed an informed written consent at the beginning of the study, and data confidentiality, voluntary participation, and the right to withdraw from the study were guaranteed.

To assess depression among the participants, the short form of the Beck Depression Inventory was employed. Four individuals scored above 16 and were excluded from the study due to severe depression. Subsequently, the remaining participants were randomly assigned into 2 groups of 16 using a random block design method.

However, 2 patients were excluded from the present study due to absence from more than 2 sessions during the class period, and another 6 were excluded from the study due to unwillingness to complete the questionnaire during the follow-up phase. Ultimately, each group was reduced to 12 patients.

The inclusion criteria included at least primary education, informed consent to participate in the study, Iranian citizenship, a definitive diagnosis of breast cancer, being sexually active, having a marital commitment and living with a spouse, no metastasis of the disease, no acute psychiatric disorders, no use of related drugs, and at least 3 months after the period of conventional cancer treatment. Exclusion criteria included not attending 2 or more sessions, divorce or death of the spouse during the study period, consumption of any sedatives, use of counseling services outside of the study during the study, diagnosis of metastasis, and severe depression. Those eligible and interested in participating in the study were informed about the purpose and how the study would be conducted, as well as its future applications. After obtaining their consent to collaborate, they were assured that completing the questionnaire would not result in any harm or damage, and it was emphasized that participation in the study was voluntary and that their information would be kept confidential.

The data collection tool included 3 demographic, women’s sexual well-being, and the short form Beck Depression Inventory (BDI-13) questionnaires:

The demographic questionnaire included: age, level of education, number of children, duration of marriage, family history of breast cancer, duration of recovery from cancer, and type of treatments.

Women’s sexual well-being questionnaire (FSWS scale): this questionnaire was prepared by Rosen et al and included 17 items and 4 factors (interpersonal relationships, the cognitive-emotional domain, physical stimulation, and satisfaction from experiencing an orgasm). The scoring of the items is based on a Likert scale from 1 to 4 from “ompletely agree” to “completely disagree.”^[13]^ In the research of Tavares et al, the reliability of the scale and subscales was estimated to be between 0.77 and 0.93 using Cronbach’s alpha coefficient.^[32]^ In Iran, the validity and reliability of this questionnaire were confirmed in the study of Chalmeh and Abdolahi, and the reliability of the scale measuring interpersonal relationships, cognitive-emotional domain, physical stimulation, and orgasm satisfaction using Cronbach’s alpha coefficient was reported to 0.79, 0.80, 0.73, and 0.67, respectively. They reported the scale total reliability as 0.82.^[33]^ Also, the obtained results showed that the correlation coefficients between the factors extracted from the women’s sexual well-being scale together and with the total score were significant (*P* < .01), which indicates the validity of this tool.

## 3. Beck Depression Inventory (BDI-13) Short Form Questionnaire

This questionnaire was developed by Beck and colleagues in 1988 and consists of 13 items designed to measure various symptoms of depression, such as emotional, cognitive, motivational, and physiological aspects. Each item has a 4-point scale ranging from 0 to 3, with the total score ranging from 0 to 39. Patients who score between 0 and 4 are considered normal; scores between 5 and 8 indicate mild depression; scores between 9 and 15 suggest moderate depression; and scores of 16 and above are classified as severe depression.^[34]^

To conduct the research, the researcher completed training courses on sexual mindfulness techniques in specialized workshops under the supervision of expert instructors. After obtaining a valid certificate and in collaboration with a psychologist holding a PhD in family counseling with 13 years of professional experience, who has led several mindfulness workshops and training programs at the university level and has extensive experience working with cancer patients, the researcher conducted sexual mindfulness training sessions in 8 ninety-minute, in-person sessions, once a week, from April to June, for the members of the intervention group.

The first to third sessions included mindfulness training through standard mindfulness exercises such as mindful breathing, diaphragmatic breathing, body scan, mountain meditation,^[35]^ and the Prevention and Relationship Enhancement Program (PREP), which focuses on the speaker-listener technique (designed to slow down communication and open space for mutual understanding) and is used for resolving marital conflicts and improving relationships.^[36]^

The fourth to eighth sessions included an introduction to the anatomy and physiology of male and female sexuality, along with teaching concepts of sexual mindfulness.^[37]^ In this technique, participants were taught to gently hold each other’s genitals and focus on their breathing to slow down the arousal process and become more aware of their breath and body sensations. Since there was no motivation to increase arousal, couples could create a low-pressure environment and focus on acceptance and awareness. The mindfulness practice in this low-arousal setting prepared couples to maintain a mindful state even if arousal increased. The goal of this exercise wasn’t to reach orgasm or experience arousal, but to learn the techniques of focus and awareness of sexual feelings throughout a sexual experience, freeing themselves from distractions and sexual expectations. Additionally, the technique of conscious hugging was taught, which involves embracing your partner with full body contact, silence, focusing on breathing, and being aware of one’s own body and that of their spouse, as well as consciously holding each other’s hands, which emphasizes love and affection.^[38,39]^ Finally, the tantric technique was introduced, focusing on massage, which not only helps couples recognize each other’s sensitive sexual points but also fosters greater relaxation and intimacy between them.^[40]^

Table 1 presents the educational content of the mindfulness sexual sessions.

**Table 1:** Program Schedule for sexual mindfulness intervention sessions.

Session	Content of sexual mindfulness intervention
Session 1	Introduction of the facilitator, participant introductions, exploration of participants’ motivations and expectations for the therapeutic process, overview of session guidelines and objectives, clarification of the number of sessions, introduction to mindfulness principles and the practice of present-moment awareness, and guided practice of mindful breathing meditation Home-based exercises: 1. Routine daily activities with mindful awareness (e.g., dressing, washing dishes, and showering) 2. Practice of the 3-min mindful breathing exercise to enhance mindfulness skills
Session 2	Body scan meditation to foster somatic mindfulness Guided diaphragmatic breathing meditation Home-based exercises: 1. Practice of the 3-min mindful breathing exercise 2. Body scan meditation 3. Diaphragmatic breathing exercises
Session 3	Guided mountain meditation to cultivate emotional resilience and introduction to the Prevention and Relationship Enhancement Program (PREP), emphasizing the speaker-listener technique to slow communication and open space for mutual understanding. This evidence-based approach is designed to enhance couple relationships, facilitate conflict resolution, and strengthen family cohesion Home-based exercises: 1. Practice of the PREP speaker-listener technique 2. Mountain meditation. 3. Daily practice of mindful awareness of pleasant experiences during their occurrence, using this awareness to support mood regulation when mood declines
Session 4	An overview of male and female sexual anatomy and physiology, discussion of gender-specific sexual needs to promote mutual understanding, Correction of misconceptions about sexual relationships Home-based exercises: 1. Daily mindfulness meditation (mountain meditation or body scan) 2. Participants were asked to use the PREP technique to communicate their sexual expectations and preferences and to discuss intrusive thoughts and anxiety-provoking rumination during sexual interactions with their spouses
Session 5	Highlighting the role of verbal communication, identifying barriers to discussing sexual matters, strategies for initiating constructive dialogue about sexual concerns, formulating requests to achieve desired relational outcomes, and fostering empathy Mindful Embrace Meditation: participants engage in a full-body embrace with their partner in silence, focusing on synchronized breathing and heightened awareness of their own and their partner’s physical presence. Holding hands, couples intentionally cultivate feelings of love and compassion toward each other Home-based exercises: 1. Daily mindfulness meditation to enhance mindfulness skills 2. Mindful embrace meditation (every night)
Session 6	Enhancement of sexual awareness, training in focused attention skills, and exploration of expectations in sexual interactions Sexual mindfulness meditation: participants are guided to gently hold each other’s genitals, maintaining awareness of breathing and tactile sensations to slow the arousal process. The objective is to eliminate preconceived expectations (e.g., orgasm-focused goals) and reduce intrusive thoughts, such as self-Criticism or body image anxiety. This practice encourages nonjudgmental presence during sexual activity, enhancing relational quality and sexual satisfaction Home-based exercises: 1. Daily mindfulness meditation to enhance mindfulness skills 2. Mindful embrace meditation (every night) 3. Practice a 10-min sexual mindfulness meditation (at least 3 times/wk)
Session 7	Education on human sexual response cycles during the caressing phase and guided body exploration to identify erogenous zones Tantric relaxation technique: conducted in a serene environment with dim lighting and calming music, this exercise involves synchronized deep breathing with continuous eye contact. Using a natural massage oil (e.g., coconut oil), partners massage each other’s bodies, including the neck, ears, back, and erogenous zones (e.g., breasts, vulva, buttocks, perineum, testicles, and penis). Couples engage in dialogue about sensations elicited by touch, focusing on areas that heighten arousal to enhance eroticism. This technique fosters relaxation, intimacy, and deeper understanding of each other’s sensitive areas Home-based exercises: 1. Daily mindfulness meditation to enhance mindfulness skills 2. Mindful embrace meditation (every night) 3. Tantric relaxation exercise (at least once per week) 4. Practice a 10-min sexual mindfulness meditation (at least 3 times/wk)
Session 8	Comprehensive review of previously taught exercises, synthesis of the entire intervention program, discussion of strategies for implementing future practices and sustaining daily exercises, allocation of time for participant questions and feedback, and administration of a post-intervention assessment using the Women’s sexual well-being questionnaire (FSWS scale) for the intervention group

FSWS = Women’s sexual well-being questionnaire.

The intervention sessions were delivered exclusively to participants in the intervention group, while their spouses were involved only in selected assigned home-based exercises.

During the sessions, the intervention group received 2 text messages to remind and encourage them to do the exercises related to each session during the week. In addition, to ensure and encourage participation in the meditation sessions, the results and feelings that each of these individuals experienced after their weekly practices were discussed at the beginning of each training session.

Meanwhile, the control group was not subjected to any planned intervention or training.

The sexual well-being questionnaires were completed by the intervention and control groups in the pretest and immediately after the intervention, and 3 months after the end of the sessions.

The content of the sexual mindfulness-based training program was developed based on a literature review, including the guidebook “Mindfulness-Based Therapy” and various other sources, and its content validity was assessed qualitatively. First, 8 experts in different fields (clinical psychologist, reproductive health specialist) who were willing to participate in the study were selected, and the content of the sessions was sent to the experts for review and comments. Finally, based on the suggestions and the opinion of the research team, the final content of the mindfulness sessions was compiled.^[22,23,41-43]^

After the intervention and data collection, all data were entered into SPSS version 24 (IBM Corp., Armonk). Independent-samples *t*-tests were used to compare baseline sexual well-being scores between the intervention and control groups. Analysis of covariance (ANCOVA) was performed to compare sexual well-being scores between the 2 groups immediately after the intervention and at the 3-month follow-up, with baseline sexual well-being scores included as a covariate. Chi-square tests were used to compare categorical variables between the 2 groups. Cohen’s *d* was calculated to estimate the effect size for between-group differences. The significance level was set at 0.05. A single-blinding process was implemented in this study. The statistical analyst was blinded to group allocation, and the intervention and control groups were coded as groups A and B, respectively.

## 4. Results

As reported in Table 2, the average age in the control group was 43.58 and in the intervention group was 44.83, which statistically had no significant difference based on the independent samples *T*-test (*P* = .68). The average length of marriage of the control group was longer than the intervention group, but it did not show a statistically significant difference (*P* = .51). There was no statistically significant difference in the number of children in both groups (*P* = .83).

**Table 2:** Determining and comparing the demographic traits of the studied groups.

Variable	Group	Number	Mean ± SE	*P* value
Age	Control	12	7.08 ± 43.58	.68*
	Intervention	12	7.31 ± 44.83	
Number of child	Control	12	2.25 ± 0.97	.83*
	Intervention	12	2.17 ± 0.97	
Marriage	Control	12	10.93 ± 22.33	.51*
	Intervention	12	9.87 ± 19.50	

* Fisher’s exact test.

As presented in Table 3, there was no statistically significant difference in education across the 2 groups, as per the rigorous Fisher test (*P* = .35). Furthermore, the highest frequency regarding the duration of the treatment in both groups was between 3 and 5 months, which statistically did not show a significant difference between the 2 control and intervention groups, and they were homogeneous (*P* = .09).

**Table 3:** Determining and comparing the duration of treatment of the studied groups.

Variable	Category	Control group N (%)	Intervention group N (%)	*P* value
Level of education	Below High School Diploma	6 (50.0%)	4 (33.3%)	35/0*
	High School Diploma	(25.0%)3	6 (50.0%)	
	Bachelor	(25.0%)3	(25.0%)3	
	Master	0.0%))0	(8.3%)1	
Duration of treatment	3–5 mo	7 (58.3%)	6 (50.0%)	.09*
	6–12 mo	(8.3%)1	4 (33.3%)	
	1–5 yr	4 (33.3%)	(0.0%)0	
	5–10 yr	(0.0%)0	(16.7%)2	

* Fisher’s exact test.

Table 4 presents the distribution of treatment types among the participants. The majority had received all of the mentioned treatments (chemotherapy, surgery, radiotherapy, and hormone therapy), and Fisher’s exact test showed no statistically significant difference between the intervention and control groups in terms of the type of treatment (*P* = .21).

**Table 4:** Determining and comparing the type of treatment of the studied groups.

Variable	Category	Control group N (%)	Intervention group N (%)	*P* value
Type of treatment	Chemotherapy	(8.3%)1	(0.0%)0	.21*
	Hormontherapy	(8.3%)1	(0.0%)0	
	surgery	(0.0%)0	(8.3%)1	
	Radiotherapy and Chemotherapy	(0.0%)0	3 (25.0%)	
	Radiotherapy and Hormontherapy and Chemotherapy	(8.3%)1	(0.0%)0	
	Radiotherapy and surgery and Chemotherapy	(8.3%)1	3 (25.0%)	
	Chemotherapyand surgery	(8.3%)1	(0.0%)0	
	All items	(58.3%)7	5 (41.7%)	

* Fisher’s exact test.

As presented in Table 5, the independent-samples *t*-test showed that there was no statistically significant difference in the mean sexual well-being score between the intervention and control groups before the intervention (*P* = .29). Immediately after the intervention, the mean sexual well-being score increased from (39.33 ± 25.56) to (56.17 ± 22.29) in the intervention group; however, the between-group difference was not statistically significant (*P* = .11). At 3-month follow-up, the mean sexual well-being score was significantly higher in the intervention group (66.42 ± 13.79) than in the control group (51.92 ± 20.62; *P* = .001).

**Table 5:** Comparison of the mean score of the sexual well-being index between the 2 groups, before the intervention, immediately after the intervention, and 3 months after the intervention.

Variable	Group	Before intervention Mean ± SD	Immediately after the intervention Mean ± SD	Three months after the intervention Mean ± SD	Within-group *P* value‡	Effect size (Cohen’s *d*)
Sexual well-being	Control	26.56 ± 50.92	15.76 ± 55	20.62 ± 51.92	.60	0.04
	Intervention	25.56 ± 39.33	22.29 ± 56.17	13.79 ± 66.42	.001	0.55
Mean difference (95% CI)		−11.60 (−33.66, 10.48)	1.17 (−15.17, 17.51)	14.5 (0.35, 29.35)		
Between group *P* value		0.29*	0.11†	0.001†		
Effect size (Cohen’s *d*)		0.44	0.06	0.83		

* Independent *T*-test.

† Analysis of covariance.

‡ Repeated measures test.

Within-group repeated measures analysis showed a statistically significant change in sexual well-being over time in the intervention group (*P* = .001), whereas no statistically significant change was observed in the control group (*P* = .60).

## 5. Discussion

The present study indicates that improving the sexual well-being of women with breast cancer can be significantly influenced by education focused on Sexual Mindfulness. These results are consistent with Leavitt et al’s study, which showed that sexual mindfulness encourages people to focus on greater intimacy and enjoyment of their sexual experience instead of trying to reach orgasm. This is accompanied by higher stimulation and arousal, which causes the sexual well-being of couples to improve significantly.^[44]^ Also, these findings were consistent with the study of Dawson et al, who stated in their research that Sexual Mindfulness, with moment-to-moment awareness during sexual activities and paying more attention to each other’s sexual stimuli and feelings allows people to allow people to their emotions and sexual feelings and through getting rid of mental and emotional distress in sexual situations, it leads to the improvement of sexual well-being in patients.^[45]^

In addition, in the present study, in the 3-month follow-up phase after the intervention, a significant increase in the level of sexual well-being was observed in the patients over time, which agreed with the results of other studies that showed mindfulness-based training requires continuous practice to effectively improve sexual performance and well-being.^[46,47]^ The possible mechanism of this positive effect can be that sexual mindfulness, by providing the necessary skills to stay aware and focus on slowing down sexual activity, increases awareness and acceptance towards the body and sexual experiences, and facilitates providing the basis for having pleasurable sexual relationships for couples.^[46,48,49]^ Sexual mindfulness empowers individuals to move away from avoiding and suppressing their sexual desires and needs, and instead engage actively and mentally with their partner during sexual encounters. This can ultimately lead to increased sexual desire and arousal, improved sexual performance, and enhanced sexual well-being of these patients.^[30,37,50]^ Additionally, sexual mindfulness can improve intimate relationships with one’s partner by reducing stress and anxiety related to the ability to perform sexually well and feel desirable. This, in turn, can have a positive impact on sexual well-being.^[25,45,51]^ These findings align with the research by Brotto, which showed that women often experience significant negative self-judgment during their sexual encounters. These stresses and mental worries can decrease libido and arousal, negatively affecting their sexual response. However, mindfulness, aimed at fostering active awareness, positively influences neurotransmitters involved in sexual function within the central nervous system, enhancing both the mental and sexual coordination of women, which correlates with increased desire, interest, and sexual intimacy.^[23]^

In Leavitt et al’s study, the results indicated that mental awareness during sex may play a more important role in women’s sexual satisfaction than men’s sexual satisfaction. The majority of women experience more anxiety during their sexual activities, with an increased focus on their partner’s arousal and orgasm. Mindfulness can reduce negative thoughts, leading to women reporting higher satisfaction from relationships and strengthening sexual well-being in these individuals.^[22]^

Improving sexual dysfunction after cancer is associated with an increased quality of life in these individuals.^[52-54]^ Further research showed that mindfulness monotherapy over 4 weeks can significantly improve sexual desire, arousal, and orgasm in women with sexual dysfunction.^[55]^ A randomized controlled trial compared mindfulness-based cognitive therapy combined with psychoeducation to standard sex therapy. The results showed that both interventions improved symptoms of sexual interest/arousal disorder. However, in the mindfulness-based approach, individuals reported higher levels of interoceptive awareness, self-compassion, and reduced self-criticism, which ultimately led to increased sexual desire and arousal.^[56]^

Based on evidence, women with cancer who underwent short-term or long-term mindfulness interventions reported high levels of desire, arousal, satisfaction, and overall better functioning in all sexual areas.^[30]^ Such mindfulness-based interventions or sexual mindfulness are of higher importance, especially in breast cancer survivors who face numerous sexual problems after conventional treatments, and the most effective medical treatments for sexual dysfunction in these patients have consumption limitations.^[57]^ Therefore, when patients are provided with information along with skill-based educational applications, it can more broadly enhance sexual health and quality of life.^[58]^

Considering that the findings of this study can aid in the recovery of women’s sexual well-being after breast cancer treatment, it is recommended that healthcare providers and therapists incorporate such interventions into their palliative care and counseling for these individuals. Additionally, sexual mindfulness can be considered a safe therapeutic model for improving various components of sexual health for different individuals and can create a context for successful sexual interactions among couples.

This study has limitations that need to be addressed in future research. In the present study, although baseline sexual well-being did not differ significantly between the intervention and control groups, a numerical imbalance was observed, with lower baseline scores in the intervention group. Given the small sample size and substantial variability in sexual well-being scores, this baseline imbalance may have influenced the magnitude of the observed changes over time. Although baseline scores were accounted for in the between-group analysis, the possibility of residual influence cannot be completely excluded. Therefore, these findings should be interpreted with caution and confirmed in larger randomized controlled trials. Future research could be carried out in other geographical areas with larger samples and different cultures longitudinally to enhance the generalizability of the current results. Our sample included only 1 person per relationship, but future research could collect couple-level data. Because sexual activity is a shared experience that relies on the presence of a partner, each person’s mindfulness may be linked to the sexual well-being and satisfaction of the other. Future research could examine the effectiveness of these interventions in other populations of women with diverse sexual issues. This could provide stronger evidence for the application of these interventions. Another limitation of this study was self-reporting bias, which could lead to an unrealistic decrease or increase in reported sexual well-being. Additionally, future research could track outcomes over longer time intervals of 6 or 12 months to assess the durability of the treatment, as its effectiveness may diminish over time.

## 6. Conclusion

Sexual mindfulness, by overcoming sexual concerns and discomforts caused by breast cancer, can enhance the various components of sexual well-being in patients. The integration of sexual mindfulness-based interventions into breast cancer care and palliative programs can be a crucial step in enhancing the quality of life and sexual well-being of these patients.

## Acknowledgments

This article is extracted from a thesis approved by Shahrekord University of Medical Sciences. Hereby, we would like to thank the esteemed Research Deputy of Shahrekord School of Nursing and Midwifery and all those who helped us conduct this study.

## Author contributions

**Investigation:** Asefeh Shirdashtzadeh Kerdabadi.

**Formal analysis:** Hadi Raeisi Shahraki.

**Methodology:** Hadi Raeisi Shahraki.

**Supervision:** Zeinab Tavakol, Fereshteh Rasti Borujeni, Zahra Davoodi.

**Writing – original draft:** Asefeh Shirdashtzadeh Kerdabadi.

**Writing – review & editing:** Asefeh Shirdashtzadeh Kerdabadi, Zeinab Tavakol, Fereshteh Rasti Borujeni.
